# The Use of Imaging Techniques in Chronic Kidney Disease-Mineral and Bone Disorders (CKD-MBD)—A Systematic Review

**DOI:** 10.3390/diagnostics11050772

**Published:** 2021-04-26

**Authors:** Ana Pimentel, Jordi Bover, Grahame Elder, Martine Cohen-Solal, Pablo Antonio Ureña-Torres

**Affiliations:** 1 Department of Dialysis AURA Nord Saint Ouen, 93400 Saint Ouen, France; anappimentel@gmail.com; 2 Fundació Puigvert, Department of Nephrology, IIB Sant Pau, RedinRen, Barcelona, 08025 Catalonia, Spain; jbover@fundacio-puigvert.es; 3 Department of Renal Medicine, Westmead Hospital, Sydney, NSW 2145, Australia; g.elder@garvan.org.au; 4 Bioscar INSERM U1132, Department of Rheumatology, Université de Paris, Hôpital Lariboisière, 75010 Paris, France; martine.cohen-solal@inserm.fr; 5 Department of Renal Physiology, Necker Hospital, University of Paris Descartes, 75015 Paris, France

**Keywords:** bone, fracture, bone mineral density, computed tomography, cortical bone, trabecular bone, CKD-MBD, dual-energy X-ray absorptiometry

## Abstract

Although frequently silent, mineral and bone disease (MBD) is one of the most precocious complication of chronic kidney disease (CKD) and is omnipresent in patients with CKD stage 5. Its pathophysiology is complex, but basically, disturbances in vitamin D, phosphate, and calcium metabolism lead to a diverse range of clinical manifestations with secondary hyperparathyroidism usually being the most frequent. With the decline in renal function, CKD-MBD may induce microstructural changes in bone, vascular system and soft tissues, which results in macrostructural lesions, such as low bone mineral density (BMD) resulting in skeletal fractures, vascular and soft tissue calcifications. Moreover, low BMD, fractures, and vascular calcifications are linked with increased risk of cardiovascular mortality and all-cause mortality. Therefore, a better characterization of CKD-MBD patterns, beyond biochemical markers, is helpful to adapt therapies and monitor strategies as used in the general population. An in-depth characterization of bone health is required, which includes an evaluation of cortical and trabecular bone structure and density and the degree of bone remodeling through bone biomarkers. Standard radiological imaging is generally used for the diagnosis of fracture or pseudo-fractures, vascular calcifications and other features of CKD-MBD. However, bone fractures can also be diagnosed using computed tomography (CT) scan, magnetic resonance (MR) imaging and vertebral fracture assessment (VFA). Fracture risk can be predicted by bone densitometry using dual-energy X-ray absorptiometry (DXA), quantitative computed tomography (QTC) and peripheral quantitative computed tomography (pQTC), quantitative ultrasound (QUS) and most recently magnetic resonance micro-imaging. Quantitative methods to assess bone consistency and strength complete the study and adjust the clinical management when integrated with clinical factors. The aim of this review is to provide a brief and comprehensive update of imaging techniques available for the diagnosis, prevention, treatment and monitoring of CKD-MBD.

## 1. Introduction and Pathophysiology

The mineral and bone disorders associated with chronic kidney disease (CKD) are often progressive in earlier stages of CKD but remain clinically silent until stages G3b-G4 (estimated glomerular filtration rate (eGFR) 30 to 44 or 15 to 29 mL/min/1.73 m^2^ of body surface). Serum bone biomarkers are the earliest indicators of mineral bone diseases (MBD) during CKD progression, starting with decreased circulating alpha klotho levels and an increase of serum fibroblast growth factor 23 (FGF23). As CKD progresses, circulating 1,25 dihydroxyvitamin D values decrease, serum parathyroid hormone (PTH) rises and subsequent detectable alterations of serum calcium and phosphate metabolism occur [1]. The prevalence of CKD is still growing above other life-style diseases, affecting over 850 million people worldwide [2], and as patients get older and have a longer life expectation, the prevalence of CKD rises, together with the incidence of MBD. The combination of CKD-related low or high rates of bone turnover, mineralization defects and reduced bone mass has been termed renal osteodystrophy. However, seen more globally, bone disease in CKD is the sum of CKD-specific risk factors, which are in turn dependent on CKD progression, together with age- or sex-related bone loss. These complex and interacting changes to bone require careful evaluation, including using imaging techniques [3].

**Figure 1:**
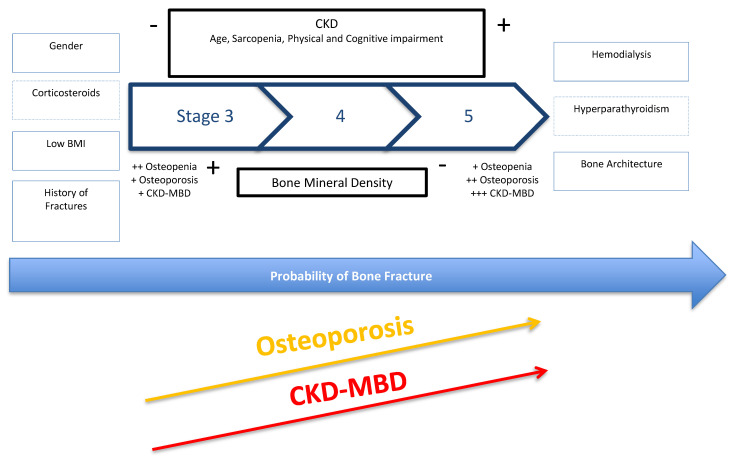
CKD progression: risks factors for bone fragility in CKD stages. In stage 3 it is more likely to have a more relevant contribution of osteoporosis to bone fragility. On the other hand, in stage 5 CKD-MBD and osteoporosis may have a more relevant role. Abbreviation: BMI, body mass index.

**Figure 2:**
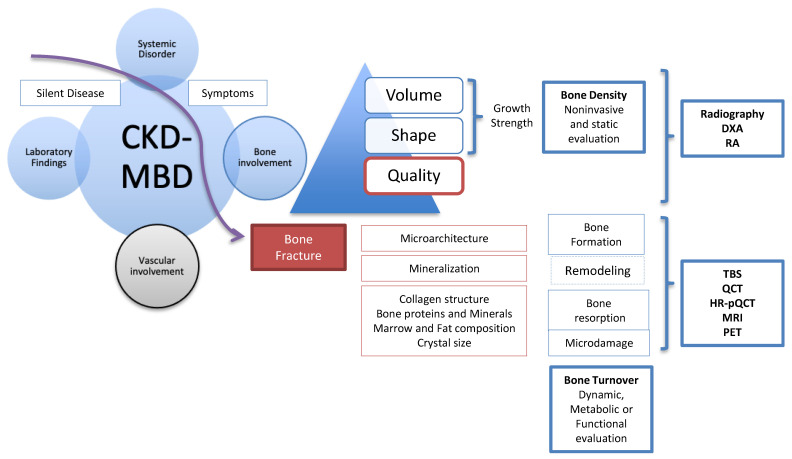
Progression of CKD-MBD from asymptomatic disease to bone fracture, bone parameters and associated imaging techniques.

In CKD stages 1 and 2 (eGFR >60 mL/min/1.73 m^2^), risk factors for bone fracture are the same as in the general population and include a history of previous fracture, female gender, older age, lower body mass index (BMI) and the use of corticosteroids (Figure 1). As the majority of these are directly related to osteoporosis risk, screening tools are similar. Similarly, in CKD stage 3b, the prevalence of osteopenia or osteoporosis is close to that of the general population. However, as CKD advances, osteoporosis prevalence increases [4] due to age-related bone loss in addition to the CKD-MBD related bone disease (Figure 1), until ‘osteoporosis’ affects the majority of patients with CKD stage 5 [5]. However, the diagnosis of osteoporosis is based solely on bone mineral density (BMD) measurement, whereas in patients with advanced CKD that diagnosis will include several additional entities and BMD limited by the presence of abdominal aorta and vertebral articular calcifications. A diagnosis of osteopenia or osteoporosis based on BMD will not discriminate between high and low bone turnover (CKD related or not) or other diseases with mineralization defects. On the other hand, the diagnosis of renal osteodystrophy (ROD) has been based on bone biopsy, and a standard classification using the indices of bone Turnover, Mineralization and Volume (TMV) was proposed, in order to improve comparison between bone biopsy studies in patients with CKD, and to improve guideline development for management and treatment. Whilst renal osteodystrophy is an integral component of CKD-MBD and contributes to an increased risk for fracture, the entity of CKD-MBD also includes biochemical abnormalities and vascular and soft tissue calcifications, which result in heightened cardiovascular risk and mortality and reinforce the complexity of the CKD patient (Figure 2) [3].

Skeletal fractures are frequent and are a major cause of CKD-related mortality and mortality in general. Severe osteoporotic fractures affect the spine, which is mainly composed of trabecular bone, and the hip and wrist, mainly composed of cortical bone. In CKD stages 1 to 3a, fractures are assumed to be osteoporotic fractures as in the general population, although they may be associated with mild secondary hyperparathyroidism (SHPT). In CKD G3b to G5, there is a 6-fold increased risk of hip fracture when compared with individuals with normal eGFR [6]. In addition, patients above 75 years with an eGFR of <45 mL/ min/1.73 m^2^ have a 2-fold increase in hip-fracture-related mortality risk [7]. Bone fractures, at any skeletal site occur more frequently in hemodialysis patients than in the general population [8,9]. In the DOPPS cohort that included a total of 36,337 patients from 12 countries, a 1.5 to 8-fold increased rate of peripheral fractures was reported, together with an increased risk of all-cause mortality [10]. The high mortality and hospitalization costs related to fracture in patients on dialysis enhances the economic burden of bone disease. After renal transplant, bone diseases remain a major cause of morbidity with a significantly higher risk of fractures as well as increased health care costs, hospitalization, and mortality [11]. An important change in the bone paradigm occurred after the publication of the recent 2017 KDIGO CKD-MBD guidelines and it is now considered “time for action”.

Skeletal fractures are a major clinical outcome, and in many cases they are easy to evaluate using standard x-ray imaging, even if they are associated with few or no symptoms. In the context of CKD-MBD, standard lateral spine x-rays should be protocolized to detect vertebral fractures concurrently with evaluating the presence or absence of abdominal aortic calcification (AAC), for which there are a number of semiquantitative scoring methods [12]. The VFA (vertebral fracture assessment), either using low radiation dose imaging of the lateral spine with a bone densitometer, is another method with moderate sensitivity and high specificity for detecting vertebral fracture [13,14]. However, the early diagnosis of a fracture that is not detected by conventional imaging techniques may be challenging and require further careful investigation. This is the case of fatigue fractures or pseudo-fractures that occur in the presence of mineralization defects. When evoked by pain, imaging techniques offer guidance towards the type of CKD-MBD and often reveal prevalent fractures or pseudo-fractures in addition to the severity assessment of bone fragility and medical intervention monitoring.

The Kidney Disease Improving Global Outcomes (KDIGO) Guidelines highlight the need for early evaluation of bone and in 2017 recommended assessing fracture risk in patients using dual-energy X-ray absorptiometry (DXA) if the results can impact treatment decisions [15,16]. Further studies are needed to determine when and what skeleton sites should best be screened for MBD. Other techniques like CT [17] or peripheral high resolution computerized tomography (HRpQCT) provide individualized data on the cancellous and cortical bone compartments, but so far they are still dedicated to research purposes and have not been demonstrated to better predict fracture risk. Moreover, a more systematic use of imaging techniques is not only required for early diagnostic but also for a better monitoring of bone disease, because early interventions may ease adverse outcomes related to bone and cardiovascular diseases [3]. The aim of this review is to provide a brief and comprehensive update of the imaging modalities available for the diagnosis, prevention, treatment and monitoring of CKD-MBD/osteoporosis complex in CKD patients.

## 2. Characteristics of Bone Structure

Eighty percent of the human skeleton is composed of cortical bone and the other 20% is trabecular bone [18]. The proportion of cortical and trabecular bone differs according to each skeletal site, so the information provided by imaging should be considered in the light of this distribution. When evoked by pain, imaging techniques offer guidance towards the type of CKD-MBD and may reveal prevalent fractures or pseudo-fractures. Imaging can also provide information on bone fragility and monitor changes after a medical intervention.

Due to its higher proportion of cortical bone, imaging of the proximal femur or femoral neck sites reflect more accurately cortical BMD, which is correlated to femoral strength. With age, cortical bone resorption accelerates, resulting in cortical thinning and increased cortical porosity. Chronic metabolic changes such as high PTH in CKD patients further enhance these features. By comparison, vertebrae are composed of 80% trabecular bone structured in a 3-dimension network, which can change faster than cortical bone because of its large surface exposed to remodeling [19]. Consequently, the rate of bone loss or bone gain is more easily captured at this site [18]. Vertebral fractures are characteristic of osteoporosis [20], and are less prone to mechanical traumatic fractures.

## 3. Bone and Soft Tissue Imaging

Medical imaging has provided a tremendous contribution to clinical decision-making [21]. Besides assessing pathological changes, the main thrust behind the development of bone imaging has been the monitoring the use of anti-osteoporotic drugs including bisphosphonates, denosumab and others. Imaging has changed the clinical diagnosis of bone fragility and contributed to the creation of treatment algorithms [21]. As mentioned before, the specific challenge in CKD patients results from the superimposition of CKD-MBD-related alterations, which influence the interpretation of imaging modalities.

Plain radiographs do not quantify bone loss, and consequently other techniques have been developed such as radiographic absorptiometry, DXA, QUS, QCT, pQCT, HR-pQCT and more recently quantitative magnetic resonance imaging (MRI). These methods facilitate an analytic approach and may improve the distinction between differing bone pathologies and fracture prediction in patients with CKD. The main limitation is the affordability of some methods, and the need for validation in CKD populations before being suggested its use in clinical practice (Table 1).

### 3.1. Conventional Radiography

**Figure 3:**
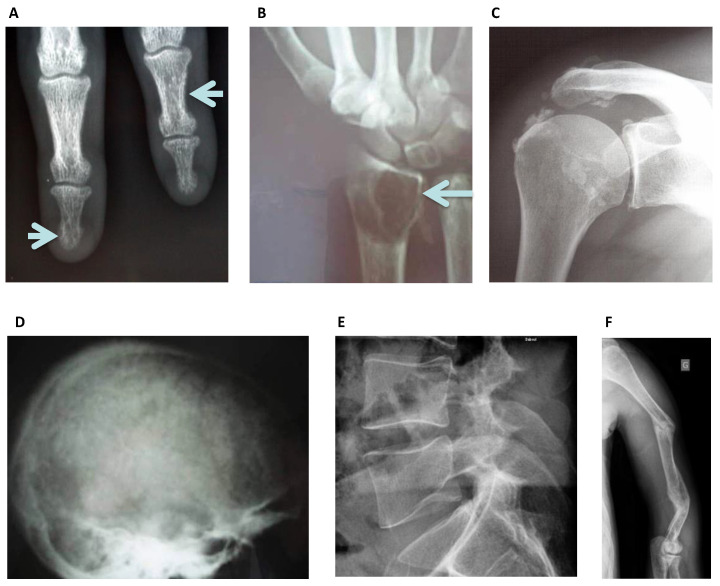
Standard Skeletal Radiography images: (**A**) Left lower arrow, sub periosteal resorption of the distal phalanx of the middle finger, and at the intermediate phalanx of the index finger as indicated by the right upper arrow in a hemodialysis patient, virtually pathognomonic of severe secondary hyperparathyroidism. (**B**) Image of a “brown tumor” at the distal radius metaphyseal as indicated by the arrow. It is a well-limited lytic lesion with endosteal scalloping, one of the possible manifestations of severe secondary hyperparathyroidism. (**C**) Periarticular calcifications of the glenohumeral ligaments, appearing as cloud-like densities that diffuse into the adjacent tenosynovial tissues. (**D**) “Salt and pepper” aspect of the calvaria seen as well-defined lucencies suggesting bone resorption. (**E**) Lateral spine X-ray can be used to assess vertebral fracture in a hemodialysis patient. (**F**) Multiple oblique spiral fractures in the proximal, middle and distal third of the humerus in a hemodialysis patient with osteomalacia.

Radiography is widely available, affordable and the most used radiological imaging method to characterize bone disease, including specific features of CKD (Figure 3). Plain x-rays can provide information about high bone remodeling and mineralization failure. In SHPT, macroscopic bone resorption is the most common finding, due to increased bone turnover promoting high osteoclast activity. The bone resorption can affect trabecular, endosteal and cortical bone envelopes, or structures close to joints located at subperiosteal, subligamentous and sub-tendinous levels. Sub-periosteal resorption is commonly found at terminal tufts of distal phalanges, subchondral resorption at acromioclavicular joints, sternoclavicular joints and sacroiliac joints. Sub-ligamentous resorption is located at the enthesis where ligaments are inserted to bone, such as at the coracoclavicular ligament and sub-tendinous resorption occurs at the femoral trochanters and ischial tuberosities [22]. In addition, skull radiographs reveal a characteristic “salt-and-pepper” appearance, with well-defined lucencies in the calvaria caused by resorption. Brown tumors are localized osteoclast tumors, identified as well-limited lucent lesions with endosteal scalloping. They are preferentially located in the pelvis, long bones or ribs. Due to their vascularized, fibrous and necrotic and liquefied tissue nucleus, changes to these lesions are a surrogate for treatment response [23]. However, these classical features of SHPT are now rarely seen worldwide.

Osteosclerosis is an additional feature of SHPT occurring predominantly in the axial skeleton and often detected on lateral lumbar spine radiographs [24] affecting the superior and inferior endplates (“rugger jersey spine”), this is mostly due to deposition of mineral crystals and calcification in the collagenous portion of the endplates. Osteosclerosis can also occur in the pelvis, ribs and clavicles [25] and these lesions may remain after the regression of SHPT. In parallel, osteoblast activation may result in new periosteal bone formation in long bones to increase cortical thickness. Of note, the periosteal reaction is separated from the cortex by a linear lucency [25].

Plain radiographs can also reveal early features of osteomalacia before fracture occurrence. These pseudo-fractures or ‘Looser-Milkman zones’ appear as linear radiolucent bands perpendicular to the cortex and are incomplete fractures. The pubis, femoral neck, scapulae, ribs and long bones are their main location, corresponding to areas of mechanical stress and the entry of blood vessels [25]. Skeletal deformations resulting from fractures of the spine, ribs and long bones may be observed in any CKD-related bone pathology.

Conventional x-rays also identify extra-skeletal calcifications. Periarticular calcifications appear as cloud-like densities that can diffuse into the adjacent joint or tenosynovial tissues, sometimes leading to erosions of adjacent bones and predisposing to fracture [25]. Visceral calcifications are often due to an inflammatory response and are rarely seen in plain radiographs, except when they are of large volume. Chondrocalcinosis may occur in fibrocartilage and hyaline cartilage in large joints including knees or shoulders. Finally, arterial calcifications can be seen as either patchy calcified atheromatous plaques or as ‘pipe stem’ arteriosclerosis without prominent luminal involvement [25]. KDIGO guidelines suggest that a lateral abdominal radiograph can be used to detect the presence or absence of vascular calcifications as reasonable alternatives to computed tomography-based imaging, and that it is reasonable to use this information to guide the management of CKD-MBD [26]. Both the Kauppila (lateral abdominal X-ray) [12] and the Adragao (hands and pelvis X-rays) calcification-scores are validated means to assess cardiovascular risk in patients with CKD [27].

**Figure 4:**
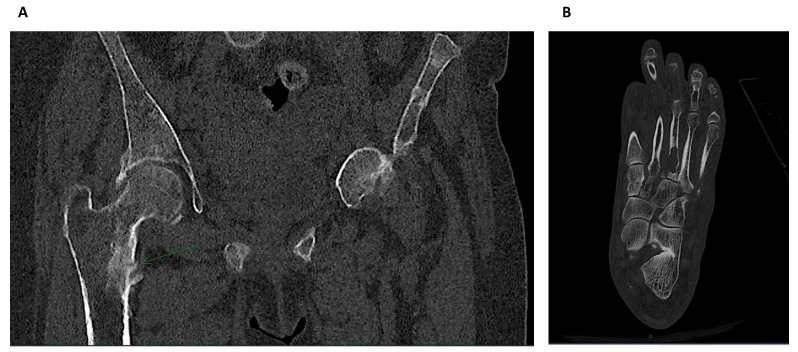
Computerized tomography images: (**A**) The arrow shows a femoral metaphyseal stress fracture distal to the lesser trochanter in a hemodialysis patient with osteomalacia. Computerized tomography is mainly used to diagnose nonobvious fractures that can allow a non-surgical approach. (**B**) Image of a “brown tumor” in the proximal zone of a metatarsal bone.

Plain radiographs may also reveal the now rare entity of amyloid arthropathy, characterized by subchondral erosions [28], often located in periarticular bone and at the site of ligamentous insertions [29]. Advanced amyloid deposition is suggested by soft tissue swelling and lytic lesions with sclerotic margins within cortical or medullary bone. The main differential diagnosis is brown tumors associated with hyperparathyroidism. Computerized tomography is mainly used to diagnose nonobvious fractures or lesions that cannot be seen by conventional x-ray (Figure 4).

### 3.2. Dual-Energy X-ray Absorptiometry

DXA is valuable for the quantification of BMD and the evaluation of fracture risk [30]. Axial measurement of the lumbar spine and hip (central DXA) using a stationary scan table is the most common modality. The technique produces little radiation and obtains a rapidly acquired two-dimensional (areal) image with good resolution. The WHO international reference standard for osteoporosis diagnosis is a DXA T-score of −2.5 or less at the femoral neck. Osteoporosis may be diagnosed in postmenopausal women and in men age 50 and older if the T-score at the lumbar spine, total hip, or femoral neck is −2.5 or less. An important limitation of DXA is the potential interference from surrounding calcifications, which may reduce the accuracy of areal BMD assessment. Hence, aortic calcifications, ligamentous calcification, degenerative changes and scoliosis will all spuriously increase BMD at the lumbar spine, leading to an overestimate of vertebral BMD.

With additional software, DXA BMD evaluation can also be combined with vertebral fracture assessment (VFA), performed with lower radiation exposure than standard X-ray. Using VFA, more patients with increased risk of fracture are identified than with DXA alone [26] and vertebral fractures can be detected in 14% of patients with normal BMD [31]. The International Society for Clinical Densitometry (ISCD) recommends using VFA as a densitometric spine imaging to detect vertebral fractures when the T-score is <−1.0 and if one or more of the following is present: women ≥ 70 years or men ≥ 80 years of age, historical height loss >4 cm (>1.5 inches), self-reported but undocumented prior vertebral fracture, or glucocorticoid therapy equivalent to ≥5 mg of prednisone or equivalent per day for ≥3 months. Whole-body DXA can also be used to evaluate total body composition (including fat mass, lean tissue mass and visceral adipose tissue mass), which can be used in the assessment of cardiovascular risk [32].

In patients with CKD, DXA BMD is a measurement of both cortical and trabecular bone at each individual site. A low BMD by DXA may indicate any combination of osteopenia / osteoporosis and superimposed renal bone disease, but can nevertheless be used as a baseline measure at the commencement of the treatment and to monitor treatment response over time [30]. ISCD guidelines regarding VFA are applicable to patients with CKD G4–G5D [15] and lateral DXA can also incorporate aortic calcification assessment for AAC scoring. All-cause mortality and cardiovascular mortality are higher in patients on dialysis and following transplantation having higher AAC scores [33,34].

BMD is predictive of fracture in CKD G3a to G5 when obtained by measuring distal radius, femoral neck, femoral trochanter or total hip DXA [35,36,37]. The association between DXA at ultradistal radius and bone histomorphometry parameters was demonstrated in 16 patients with CKD stage 3–4 and in dialysis patients [38]. Low BMD in dialysis patients is also associated with an increased risk of arteriosclerosis, coronary and vascular calcification [39] and when measured by whole-body DXA is also associated with an increased incidence of cardiovascular disease and all-cause mortality [40]. In addition, a post hoc analysis of 426 incident dialysis patients (median age 56 years, 62% men) revealed low head and pelvis BMD, and low total BMD, as assessed by whole-body DXA, were independent predictors of increased risk of all-cause and CVD mortality [41]. BMD measured at total hip and ultradistal radius sites is generally lower in dialysis patients [42] than in the general population. However, BMD at the lumbar spine is often similar to that in non-CKD patients [43], probably due to the increased prevalence of vascular calcification [43], the development of degenerative artefact in both CKD and non-CKD elderly populations and also by a potential positive effect of a milder degree of hyperparathyroidism.

BMD does not provide information about bone turnover or architecture, which are adversely affected in CKD, and decisions to initiate treatment generally rely on additional information. Nevertheless, a very low BMD is an indicator of bone fragility and should prompt investigation of its causes, such as osteomalacia.

### 3.3. DXA-Derived Trabecular Bone Score (TBS)

The trabecular bone score (TBS) is another available and validated tool that evaluates trabecular microarchitecture through BMD measures obtained at the lumbar spine by DXA [44]. Lower lumbar spine TBS was associated with a higher risk of fragility fracture in individuals with an eGFR <60 mL/min/1.73 m^2^ similarly to individuals with an eGFR ≥60 mL/min/1.73 m^2^ [42] independently of BMD and/or other fracture clinical risk factors [45]. An advantage of TBS is that overlapping vascular calcifications and degenerative changes do not interfere with its measurement. Patients on hemodialysis have a significantly lower TBS than controls without osteoporosis, and this is independent of BMD or other covariates (1.15 ± 0.181 vs. 1.32 ± 0.123, *p* = 0.001, respectively) [46]. In 59 hemodialysis patients, TBS had a good correlation with T and Z-scores at the lumbar spine and proximal femur as measured by DXA [47]. TBS was also significantly lower (1.365 ± 0.129 vs. 1.406 ± 0.125, *p* < 0.001) in 327 kidney transplant recipients (mean age = 45.3 ± 12.4 years), when compared to 981 matched healthy individuals (mean age = 45.4 ± 12.3 years) [48]. A low TBS was associated with the risk of fracture independently of FRAX (adjusted hazard ratio per standard deviation decrease 1.55; 95%CI: 1.06–2.27) [48]. In 146 patients with CKD 5 and 5D (mean age = 48 ± 13 years) undergoing DXA at the time of kidney (*n* = 114 patients) or simultaneous pancreas-kidney transplantation (*n* = 33 patients) [49], 15% had a low TBS <1.23 and TBS did not significantly differ with sex, age or prior dialysis duration. Low TBS values (≤1.31) were associated with prevalent non-vertebral fracture, independently of femoral neck BMD. In another study, 40 kidney transplant patients (mean age = 63.8 ± 11.1 years) were matched with 77 healthy controls (mean age = 50.2 ± 16 years) 10 years after their kidney transplant surgery [50]. Although BMD remained lower in the transplant recipients, TBS values were similar between the groups suggesting that bone health might have been improved by the kidney transplantation. In general, BMD measurement alone cannot always estimate the severity of bone disease, for example, both high-turnover disease and low-turnover disease may have the same BMD parameters [51] but there is an association between low TBS and CKD reflecting trabecular micro-architecture and cortical width measured by bone histomorphometry [52], including renal transplant patients. While some data now support an association between TBS and fracture risk independent of bone density in patients with CKD, a recent European consensus on the diagnosis and management of osteoporosis in CKD G4-G5D considered that TBS, as well as other DXA-based bone texture measurements, need further evaluation before their implementation in clinical practice can be advocated [15].

### 3.4. Radiographic Absorptiometry (RA)

DXA and TBS techniques may not be widely available because of their relatively high capital cost and lack of expertise in many non-industrialized countries. In this case, RA could be an alternative approach for the evaluation of BMD. RA is both rapid and inexpensive because it does not require dedicated equipment. RA measures the second metacarpal mid-shaft BMD by using X-ray radiographs, combined with digital image processing (DIP) and a computed X-ray densitometer to improve the precision and accuracy [53,54]. Several studies have shown that RA-based BMD assessment can reliably be used for the estimation of fracture risk in post-menopausal women [55]. In hemodialysis patients, a recent study in 456 hemodialysis patients demonstrate that lower metacarpal BMD measured by DIP-assisted RA predicts the risk of osteoporotic fractures [56].

### 3.5. Quantitative Computerized Tomography

QCT allows in vivo assessment of trabecular architecture, volumetric BMD and bone size, from which BMD can be estimated. QCT also provides a functional approach to bone densitometry by measuring bone strength through biomechanical parameters. QCT imaging can disclose pathological fractures and delineate joint lesions related to amyloidosis. As QCT uses a high radiation dose in a small field of view, it can also be used to monitor bone structural changes over time, disease progression and treatment efficacy. However, In vivo applications are limited. Peripheral QCT (pQCT) limits radiation to the tibia and distal radius.

### 3.6. High Resolution-Peripheral Quantitative Computerized Tomography (HR-pQCT)

**Figure 5:**
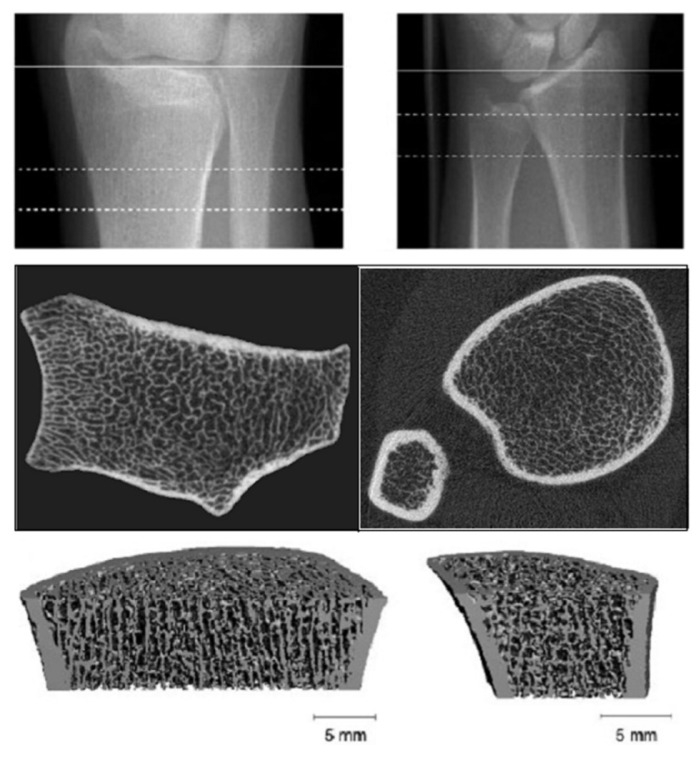
High resolution-peripheral quantitative computerized tomography (HR-pQCT): Analysis of trabecular and cortical microarchitecture proximally and distally at ulnar and radial level.

HR-pQCT provides excellent spatial resolution, differentiating trabecular from cortical bone and using a lower radiation dose (Figure 5). The distal radius and tibia contain mainly cortical bone. In patients on dialysis, HR-pQCT was more closely associated with prevalent fracture than DXA measurements [57]. HR-pQCT measures trabecular spacing, with modest limitations in measuring trabecular number and thickness [17]. In a cross-sectional study of patients on hemodialysis, women in particular were found to have significant cortical microarchitectural deterioration and abnormal trabecular parameters, compared to a normal matched population [58]. Bone microarchitecture alterations have been associated with the severity of SHPT [52], while other studies demonstrated significant cortical bone loss but no significant changes in trabecular density or microarchitecture in CKD stages 2 to 5 assessed by HR-pQCT at the distal radius [42]. Cortical impairment is reported to be associated with biochemical bone turnover markers, and may assist in identifying CKD patients at risk of fractures [14].

Bone loss in CKD is partially due to cortical bone deterioration [42] through augmentation of the cortical porosity and thinning secondary to trabecularization of the endocortical junction [5]. Changes at sites of predominant cortical bone may be a better determinant of the ‘bone disease status’ than trabecular bone-rich sites. In a longitudinal study that included 53 patients with CKD G2 to G5D, CKD patients assessed by HR-pQCT at the distal radius had rapid cortical bone loss during 1.5 years of follow-up, with declines in cortical area, density, and thickness and increases in porosity: −2.9% (95% CI −3.7 to −2.2), −1.3% (95% CI −1.6 to −0.6), −2.8% (95% CI −3.6 to −1.9), and +4.2% (95% CI 2.0 to 6.4), respectively [42] whereas trabecular bone loss was not found. In patients with CKD G2-G5D, TBS was independently associated with trabecular measures at the radius and with cortical measures at the tibia by HR-pQCT [52] and was associated with trabecular structural parameters assessed by the ‘gold standard’ of bone biopsy.

Fourteen patients undergoing kidney transplantation (*n* = 12) and parathyroidectomy (*n* = 2) were evaluated in terms of histomorphometry by iliac crest bone biopsy and micro-computed tomography on the core sample [59] showing a deterioration of cortical microarchitecture despite predominantly normal trabecular parameters. By histomorphometry analysis, high bone turnover was present in half the patients. Nevertheless, HR-pQCT requires expensive equipment not readily available for clinical use and it is still confined to examination of the distal forearm and leg. Additional limitations include individual length difference when the radial or tibial bone are evaluated longitudinally [17].

### 3.7. Magnetic Resonance Imaging (MRI)

**Figure 6:**
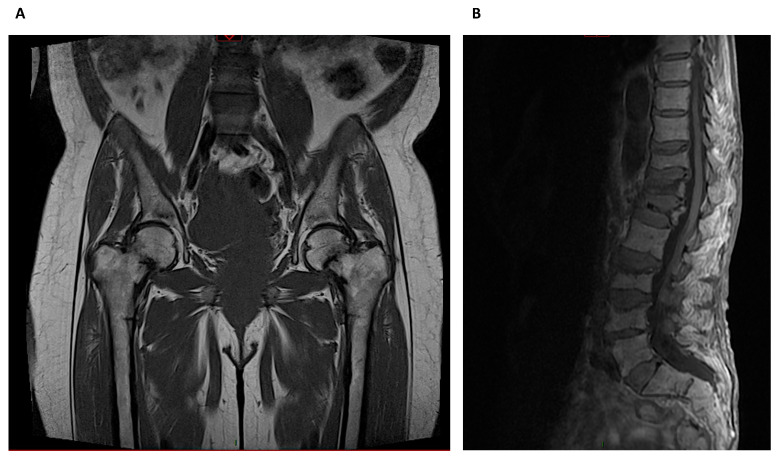
Magnetic Resonance Imaging: (**A**) Bilateral metaphyseal femoral fractures distal to the lesser trochanter with reactional associated bone edema in a hemodialysis patient with osteomalacia. (**B**) Osteoporotic lumbar spine compression fracture at T11, T12, L1, L3 and L4 vertebral body that is hyperintense on T2 and shows vivid contrast enhancement with paravertebral soft tissue edema.

Whereas HR-pQCT is more limited to peripheral skeleton regions like the radius and tibia, MRI can also image sites such as the proximal femur, but usually with lower spatial resolution (Figure 6). It was used in the past for imaging trabecular architecture at the distal radius, distal tibia and calcaneus, using photonic absorptiometry with iodine-125 (I-125) and this was subsequently replaced by dual photonic absorptiometry using gadolinium-153, and employed to study the axial skeleton (hip, spine and whole skeleton) [60]. Although MRI does not use ionizing radiation, it has largely been replaced by HR-pQCT due to rather complicated scan protocols not routinely available. Its main advantages are the direct acquisition of images in any plane and acquisition of functional information from bone and bone marrow, beyond the mineralized component [17,61]. More recently, it has been used to quantify cortical water and to differentiate bound water, a characteristic of collagen, from free water, which is characteristic of cortical porosity. Another application is imaging of marrow fat content and composition, marrow perfusion, and marrow molecular diffusion [17]. Bone marrow fat measurement by magnetic resonance spectroscopy (MRS) in eight CKD patients was 13.8% (95% CI 8.3–19.7) higher at L2–L4 when compared to matched controls (age, sex and race), with no relation to variation in PTH [62]. Marrow adiposity is higher in the lumbar spine of those with moderate to late CKD stages, compared to those with normal kidney function.

As described above, amyloidosis may cause articular and periarticular erosion, resulting in subtle radiographic signs, but amyloid deposits can be easily visualized directly on MRI [63]. Advanced MRI techniques that allow a high spectral resolution such as diffusion, perfusion and spectroscopy will most likely provide useful additional information in the future.

### 3.8. Other Imaging Techniques

**Figure 7:**
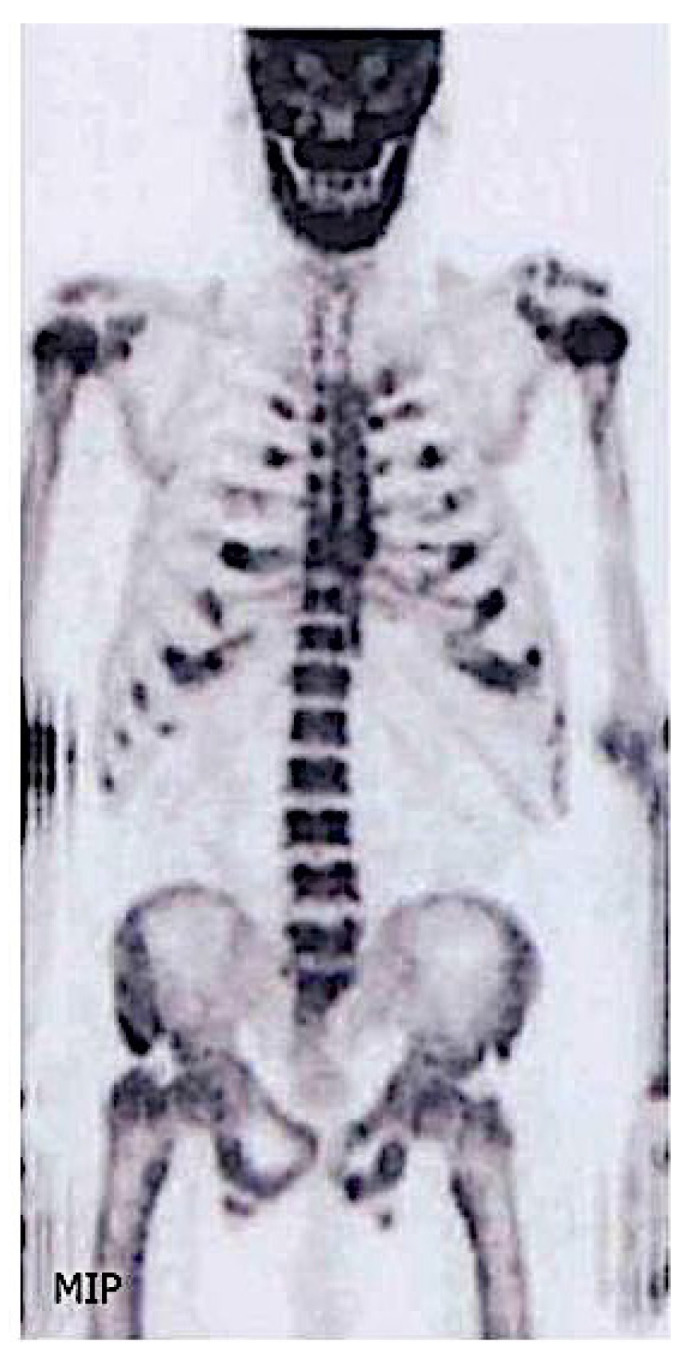
Bone scintigraphy: skeletal uptake of ^99m^Tc-labelled diphosphonate in a patient with hyperparathyroidism. There is increased bone turnover shown by increased symmetric tracer uptake throughout the skeleton, including calvaria, mandible, humeral head, accentuating the contrast between bone and soft tissue, and a beading pattern at the costochondral junctions.

Nuclear imaging techniques based on radiotracer accumulation can be used for determining the extent, progression and for monitoring of systemic diseases. Depending on the radiolabeled tracer, several diseases may be identified; we will highlight three tracers, ^99m^Tc-penta-DMSA, ^123^I serum amyloid protein (SAP) and ^131^I-β2-microglobulin (β2M). Regarding bone scintigraphy [64], skeletal uptake of ^99m^Tc-labelled diphosphonate depends primarily upon osteoblastic activity, and to a lesser extent, skeletal vascularity [65]. Patients with CKD-MBD with increased bone turnover have increased symmetric tracer uptake throughout the skeleton, including calvaria and mandible, accentuating the contrast between bone and soft tissue (Figure 7). It is also common to have a beading pattern at the costochondral junctions, particularly at the sternum. Patients with osteomalacia may present with the same pattern but may also show pseudo fractures. The bone scan is particularly sensitive for identifying rib pseudo fractures, where conventional radiology cannot detect them [65]. Globally, when compared to plain radiography, body scan seems to be more sensitive for detecting changes of MBD. Osteosclerosis can also be seen, as linear areas of increased tracer uptake in the vertebral cortical borders. Bone scintigraphy is not useful for the diagnosis of osteoporosis, but it may assist in determining if a vertebral collapse is relatively recent or longstanding.

^123^I SAP binds to amyloid deposits, and detects A, L and transthyretin amyloidosis with high sensitivity and specificity. It determines the extent and distribution of amyloid, especially in visceral organs such as liver, spleen or kidneys, but has lower potential to assess cardiac involvement [66]. Adverse events are uncommon with nuclear imaging, but it is expensive and not readily available. The ^131^I-β2M tracer can be used to identify the precursor protein of Aβ2M type amyloidosis in hemodialysis patients. The major advantage of the use of ^131^I-β2M scanning in hemodialysis patients is its high specificity; however, the tracer does not identify inflammatory changes in joints and in short-term hemodialysis patients [67].

Positron energy tomography (PET) scanning is a noninvasive quantitative imaging technique that estimates bone turnover [68] through the measurement of fluoride activity in the bone. Bone formation rate [69], osteoclast, osteoblast, erosion and mineralized surfaces correlate with the tracer intake. PET was superior to PTH in differentiating patients with low from high bone turnover in 26 hemodialysis patients [68].

Ultrasound (US) velocity at the tibia was found to be significantly lower in 42 hemodialysis patients when compared to the control group indicating cortical deterioration related to the degree of SHPT [70]. More studies are needed to validate US as a screen and diagnosis tool regarding the evaluation of CKD-MBD.

Finally, HR-MRI, Raman spectroscopy, Fourier transform infrared spectroscopy, and quantitative backscatter electron imaging [71] are also currently being used in research studies. To date, none of these techniques can be recommended for use in clinical care.

## 4. Cardiovascular Calcifications

In terms of cardiovascular calcification, there are four main described lesions in CKD patients: (1) intimal calcification associated with the atherosclerotic process; (2) medial calcification; (3) valvular calcification; (4) uremic calcific arteriolopathy (Table 2). In patients with CKD, medial calcification and intimal calcification often coexist in coronary arteries, peripheral arteries, and the aorta [72] and contribute to the high incidence of cardiovascular disease and mortality [73].

The coronary artery calcification (CAC) score is a validated and easily accessible by CT scan. It plays an important role in cardiovascular risk stratification, showing a significant association with the medium and long-term occurrence of major cardiovascular events in CKD patients, who as a group have much higher coronary artery calcium scores compared to the general population [74]. The Agatston (surface) calcium score became the gold standard endpoint for trials evaluating change in vascular calcification and, recently, a volumetric CAC score is being increasingly used because of a potentially higher sensitivity [75]. The CRIC (Chronic Renal Insufficiency Cohort) trial [76] confirmed the association between the CAC and a composite cardiovascular outcome (myocardial infarction, heart failure, and stroke).

Abdominal aortic calcification (AAC) is independently associated with cardiovascular events in the general population, but most importantly in hemodialysis patients [77]. The Kauppila score, viewed on a lateral lumbar spine plain radiograph or with VFA [14], is a semiquantitative scoring method that attributes an ordinal value to calcification (0 to 3) at 8 sites along the abdominal aorta (total maximal score 24) [12] serving as a prognostic indicator for cardiovascular mortality and all-cause mortality in patients on maintenance hemodialysis (hazard ratio, 2.39; 95% confidence interval, 1.01 to 5.66; *p* < 0.05) [77].

The Adragão score involves a semiquantitative scoring of linear calcifications using plain X-rays of the pelvis and hand and is the sum of the presence or absence of linear calcification in each section; absence 0, unilateral 1 point or bilateral 2 points [78]. It analyses calcification of the iliac, femoral, radial, and digital arteries.

Depending on resources, routine screening for cardiovascular calcification in CKD patients is controversial [79] because of the lack of specific therapies proven to reverse or attenuate vascular calcification in this population. Nevertheless, cardiovascular calcification assessment should be performed in order to predict and modify clinical outcomes especially in dialysis patients where some of the treatments used for CKD–MBD may enhance vascular calcification progression [80]. The KDIGO guidelines propose an assessment of vascular calcification in patients where it could modify therapeutic options [26]. Quantitative and/or qualitative knowledge of cardiovascular calcification could help to optimize economic resources and to assign more expensive treatments to the patients with greater expectations of improving their outcomes [81].

Finally, as vascular calcification is a product of an inflammatory process and can also promote inflammation, the combination of PET/MRI should be considered when exploring its pathophysiology [82,83,84].

## 5. Conclusions

The more advanced CKD is the less consensus there is about CKD-MBD evaluation and management. A more systematic use of imaging may assist in minimizing fracture occurrence, further bone loss and calculating individual fracture risk. Early and systematic identification of patients at risk may facilitate improved surveillance and timely interventions that could ease the burden of bone fractures and cardiovascular disease [3]. The increased availability of circulating biomarkers, in conjunction with old and novel quantitative imaging techniques and software tools to process and analyze the images may also improve the management of CKD-MBD. Combining the expertise of clinicians from various medical disciplines appears crucial to the more successful prevention of fracture in these patients.

## Figures and Tables

**Table 1:** Different imaging techniques, underlying lesion mechanisms and localization and CKD stage in which it is mostly used.

Type	Mechanism	Skeletal Site	Type of Bone Disease	CKD Stage
Plain Radiography	Bone resorption lesions Bone cysts Fractures	Sub-periosteal Subchondral Sub-tendinous Extra-skeletal calcifications All skeleton	Secondary Hyperparathyroidism Multiple Myeloma Amyloidosis Osteonecrosis Osteoporosis Calcific Uremic Arteriolopathy	All
DXA	Areal BMD measurements	Hip, distal radius, lumbar spine, whole body	Osteopenia Osteoporosis	All
Vertebral Assessment Fracture (VAF)	Vertebral deformities	Spine	Vertebral fractures	All
HR-pQCT	Trabecular architecture Volumetric BMD	Hip, distal radius, distal tibia	Secondary Hyperparathyroidism	All and research
Bone Scintigraphy	Tracer accumulation occurs in osteoblastic activity, and to a lesser extent, skeletal vascularity; Systemic amyloid burden;	Whole body	Osteoarthritis Metabolic Bone Disease: -Hyperparathyroidism and vitamin D deficiency -Osteomalacia; Fractures Enthesopathies Osteonecrosis Rare Osteoarticular Diseases: Sarcoidosis with bone involvement; Amyloidosis: ^123^I SAP scintigraphy if available—assess amyloid deposition in liver, spleen, kidneys, adrenals, localized soft tissue deposits and bones ^131^I-β2M amyloidosis	3–5
MRI	Cortical porosity Marrow fat content and composition Marrow perfusion, and molecular diffusion	Distal radius, distal tibia, calcaneus, hip, spine Whole skeleton	Secondary Hyperparathyroidism	Research
PET	Bone formation rate, osteoclast, osteoblast, erosion and mineralized surfaces	Lumbar region	Low or high bone turnover disease	All
US	Cortical deterioration	Tibia	Secondary Hyperparathyroidism	Research

DXA, Dual-energy X-ray Absorptiometry; BMD, Bone Mineral Density; HR-pQCT, High Resolution-peripheral Quantitative Computerized Tomography; MRI, Magnetic Resonance Image; PET, Positron Energy Tomography; US, Ultrasounds Velocity.

**Table 2:** Major imaging techniques for the exploration of cardiovascular calcifications.

Vascular Involvement	Clinical Imaging	Research Imaging	Clinical Outcomes
Carotid calcification	Echography and Döppler CT scan	Pulse wave velocity	Stroke Arterial stiffness
Agatston CAC score and Volume CAC score	CT scan Multi-slide CT Electron beam CT	PET scan	CV mortality and all-cause mortality Atherosclerotic events Stroke
AAC	Plain radiography CT scan Vertebral Assessment Fracture	-	Iliofemoral: renal graft failure Arterial stiffness
Valvular Calcification	Echocardiography and Döppler	-	Aortic stenosis Mitral stenosis
UCA and other calcifications	Plain radiography Echography and doppler	-	Peripheral arterial disease Arterio-venous fistula failure
Cardiac valves Coronary arteries Central and peripheral arteries	PET/MRI	PET/MRI	Detection of microcalcification within the aortic valve, great vessels, and vulnerable coronary plaque

AAC, Abdominal Aortic Calcification; CAC, Coronary Artery Calcification; CT, Computerized Tomography; UCA, Uremic Calcific Arteriolopathy.

## Data Availability

Not applicable.
